# Coordination Polymer Based on a Triangular Carboxylate Core {Fe(μ_3_-O)(μ-O_2_CR)_6_} and an Aliphatic Diamine

**DOI:** 10.3390/molecules29092125

**Published:** 2024-05-03

**Authors:** Vladimir A. Bushuev, Natalia V. Gogoleva, Stanislav A. Nikolaevskii, Sergey V. Novichihin, Dmitriy S. Yambulatov, Mikhail A. Kiskin, Igor L. Eremenko

**Affiliations:** 1 Kurnakov Institute of General and Inorganic Chemistry, Russian Academy of Sciences, 31 Leninsky Prosp., 119991 Moscow, Russia; vbushuev05@gmail.com (V.A.B.); gogolevanv@inbox.ru (N.V.G.); sanikoil@inbox.ru (S.A.N.); m_kiskin@mail.ru (M.A.K.); ileremenko@yandex.ru (I.L.E.); 2 Higher School of Economics, National Research University, 101000 Moscow, Russia; 3 N. N. Semenov Institute of Chemical Physics, Russian Academy of Sciences, Kosygina Str. 4, 119991 Moscow, Russia; s-novichihin@mail.ru

**Keywords:** iron complexes, carboxylate complexes, coordination polymers, aliphatic diamines, molecular structure

## Abstract

Interaction of the pre-organized complex of iron(II) trimethylacetate and 1,10-phenanthroline (phen) [Fe_2_(piv)_4_(phen)_2_] (**1**) (piv = (Me)_3_CCO_2_^−^)) with 1,6-diaminohexane (dahx) in anhydrous acetonitrile yielded a 1D coordination polymer [Fe_3_O(piv)_6_(dahx)_1.5_]*_n_* (**2**) and an organic salt of pivalic acid (H_2_dahx)(piv)_2_ (**3**). The structure of the obtained compounds was determined by single-crystal X-ray diffraction analysis. The phase purity of the complexes was determined by powder X-ray diffraction analysis. According to the single-crystal X-ray analysis, coordination polymer **2** is formed due to the binding of a triangular carboxylate core {Fe_3_(μ_3_-O)(μ-piv)_6_} with an aliphatic diamine ligand. Thermal behavior was investigated for compounds **1** and **2** in an argon atmosphere.

## 1. Introduction

Coordination polymers are formed by binding metal ions or metal-containing cores with bridging ligands, whose variation in structure-forming parameters allows for control over the polymer’s structure [1,2,3,4,5,6,7,8,9,10], as well as luminescent [11,12,13,14], sorption [4,15,16,17,18], magnetic [19,20,21,22], and other properties. Iron(II) coordination polymers exhibit selective catalytic activity in the hydroboration of aldehydes and ketones [23], the oxidative functionalization of alkanes [24], and in dimerization reactions of aromatic halogen derivatives [25]. Mixed-valent derivatives with a metal core {Fe^II^Fe^III^Fe^III^O} can be used in the creation of molecular switches [3,26,27].

Aliphatic diamines, which are starting compounds for polyamides, are widely used in textile and machine engineering and are therefore among the most commercially available bridging ligands can be used to form coordination polymers [28,29,30,31,32,33,34,35,36]. Metal complexes with aliphatic diamines are particularly interesting for studying their structure in crystals [30,32,33,34,36,37,38,39,40], developing bistable systems [19,28,29,41,42], and creating materials with almost zero thermal expansion effect [35].

1,6-Diaminohexane (dahx), an aliphatic diamine discovered over 100 years ago [43], is manufactured on a million-ton scale for a wide range of applications, like the production of ubiquitous polymers such as Nylon-66 (polymerization with adipic acid) [44]. According to the Scopus database, research on 1,6-diaminohexane is rapidly growing, with the number of publications doubling between 2000 and 2023. About 50% of publications are from the BRICS countries–the world’s fastest growing economies—which is evidence of 1,6-diaminohexane’s high importance in industrial applications.

Metal complexes with 1,6-diaminohexane are at the forefront of research due to their unique physical properties. Recently, work was published on the synthesis of perovskites based on antimony and bismuth halides linked by hexamethylenediamine bridges;, the authors found unique optoelectronic characteristics [45]. A study on pivalate complexes of cobalt(II) and chromium(III) with 1,6-diaminohexane was published, where the authors obtained components for molecular machines such as rotaxanes and molecular shuttles [46]. It was also shown that based on pentaiodobismutate linked by 1,6-diaminohexane bridges, it is possible to create a molecular ferroelectric semiconductor, and it is also possible to create its thin films [47]. Last year, a work was published where layered lead perovskites were studied, in which the layers were formed with protonated 1,6-diaminohexane bridges [48]. 1,6-diaminohexane can be used to obtain self-assembled hybrid layered molybdates [49], photo-electroactive indium and antimony chalcogenides [50], dynamic organic–inorganic hybrid [H_2_dahx]ZnBr_4_ crystals with the ability to control phase transitions [51], and hybrid organic–inorganic borate frameworks [52].

In our previous work, we showed that the interaction of cobalt(II) pivalate with 1,6-diaminohexane led to the formation of a 1D coordination polymer [Co(piv)_2_(dahx)]*_n_*, exhibiting a structural phase transition below 205 K, which was followed by a change in the coordination environment of cobalt(II) ions, the network of intra- and intermolecular hydrogen bonds, and the packing density of molecules in the crystal [19].

Despite the long history of using 1,6-diaminohexane to synthesize coordination compounds, according to the Cambridge Structural Database (CSD version 5.45 (November 2023)), only 34 compounds containing structure-directing fragments in which any metal is connected with a nitrogen atom of 1,6-diaminohexane are known, and to the best of our knowledge, there are no previously structurally characterized molecular complexes or coordination polymers of iron with 1,6-diaminohexane. Moreover, the CCDC database does not contain data on other structurally characterized iron polymers, where aliphatic diamine ligands, including 1,4-diaminobutane, 1,5-diaminopentane, 1,7-diaminoheptane, 1,8-diaminooctane, 1,9-diaminononane, and 1,10-diaminodecane, could also act as flexible bridging ligands.

Building upon previous research, the purpose of the current work is to study the reactivity of 1,6-diaminohexane towards the complex of iron(II) pivalate with 1,10-phenanthroline (phen). As a result, a new coordination polymer with an {Fe_3_(μ_3_-O)(μ-piv)_6_} core was synthesized and its crystal structure and thermal properties were studied.

## 2. Results and Discussion

### 2.1. Synthesis and Characterization of ***1**–**3***

The interaction of polymeric cobalt(II) trimethylacetate (pivalate) {Co(piv)_2_}_n_ in anhydrous acetonitrile with 1,6-diaminohexane led to the formation of the coordination polymer [Co(Piv)_2_(dahx)]_n_ [19], the hydrogen bonds in which are changed under the influence of temperature, leading to control over the magnetic characteristics. Iron(II) pivalate is a coordination polymer that is extremely unstable to atmospheric oxygen and is poorly soluble in acetonitrile and ethanol. Our multiple attempts to induce the direct reaction of iron(II) trimethylacetate and 1,6-diaminohexane in an acetonitrile medium and an inert atmosphere led to the formation of a mixture of products from which we were unable to isolate individual substances. Therefore, a two-step approach was elaborated. Firstly, a pre-organized carboxylate complex with a well-studied bidentate ligand was synthesized to break the coordination polymer chains. This product was then isolated, structurally characterized, and checked for phase purity. Subsequently, the pre-organized complex was treated with 1,6-diaminohexane to obtain the desired polymer complex. The mechanism of this reaction, involving either substitution of the bidentate ligand or the formation of a heteroleptic complex, remained uncertain due to the possibility of both pathways [29,30,53,54]. We previously proposed a similar synthetic protocol to obtain 3d–4f heterometallic carboxylate complexes with N-heterocyclic carbenes [55] and phosphine ligands [56].

**Scheme 1:**
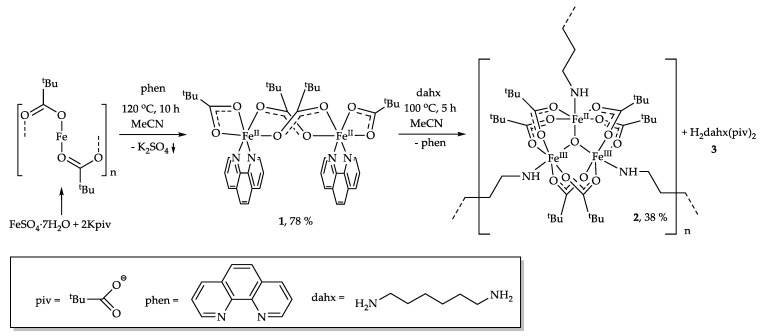
The formation of complexes **1**, **2** and an organic salt **3**.

The interaction of equimolar amounts of iron(II) pivalate and 1,10-phenanthroline in anhydrous and degassed acetonitrile led to the formation of the product [Fe_2_(piv)_4_(phen)_2_] (**1**); the addition of 1,6-diaminohexane to complex **1** led to the formation of coordination polymer [Fe_3_O(piv)_6_(dahx)_1.5_]*_n_* (**2**) and organic salt (H_2_dahx)(piv)_2_ (**3**) (Scheme 1). Complexes **1** and **2** were isolated from the reaction mixture in the form of blue–green and red crystals, respectively.

The formation of complex **2** upon the addition of 1,6-diaminohexane to the pre-organized complex is not readily apparent, considering the inert reaction atmosphere. We hypothesize the existence of a reaction of disproportionation and reduction of iron(II) in the presence of 1,6-diaminohexane, as was shown in [57], where 1,6-diaminohexane acted as a reducing agent without hydrogen in relation to the initial iron {Fe[N(SiMe_3_)_2_]_2_}_2_ complex. This assumption in our case is supported by the phase purity of the resulting compounds, the formation of pivalic acid, and its binding with 1,6-diaminohexane to form a dication–anion organic salt. In [27], a mixed-valent pivalate iron complex was obtained by controlling the volume of oxygen, since its excess led to the formation of the iron(III) product.

The fact that a chelated bidentate 1,10-phenatroline ligand is replaced by a monodentate-linked amino group seems unlikely under these conditions. We assume that this reaction occurs as a result of the oxygen traces present in the reaction mixture during crystallization and the further partial oxidation of iron(II) ions, and the formation of an oxo-carboxylate metal core is accompanied by the decoordination of 1,10-phenathroline molecules. This assumption is indirectly confirmed by the appearance of an oxo group in the final product. We emphasize the fact of partial oxidation, since, as a rule, the oxidation of iron(II) carboxylates occurs with the formation of iron(III) oxo-carboxylates, but the lack of oxygen as an oxidizing agent produces a mixed-valent complex with a yield of 38%. The decoordination of 1,10-phenatroline suggests the formation of a more stable iron(II/III) complex with two or three chelating ligands in the coordination sphere, which are more soluble n acetonitrile than the coordination polymer, and we could not isolate them from the mother liquor.

The IR spectrum of compound **2** (see Supplementary Materials, Figure S2) contains characteristic absorption bands at 3370, 3263, and 3134 cm^−1^ of stretching vibrations [58], and at 1596 and 890 cm^−1^ of bending vibrations of the amino group of primary amines [32,38]. There is a characteristic absorption of stretching vibrations of the C-N bond at 1221 cm^−1^ [30]. It is worth noting that in the IR spectrum of organic salt **3** (see Supplementary Materials, Figure S3), there are no absorption bands corresponding to the free amino group of primary amines, while strong absorption is observed at 1525 (δ_as_(NH_3_^+^) and 1482 cm^−1^ (δ_s_(NH_3_^+^), corresponding to vibrations of the ammonium group (R-NH_3_^+^) [59].

### 2.2. Crystal Structures of ***1**–**3***

The structure of compounds **1**–**3** in crystals was determined using single-crystal X-ray diffraction analysis.

**Figure 1:**
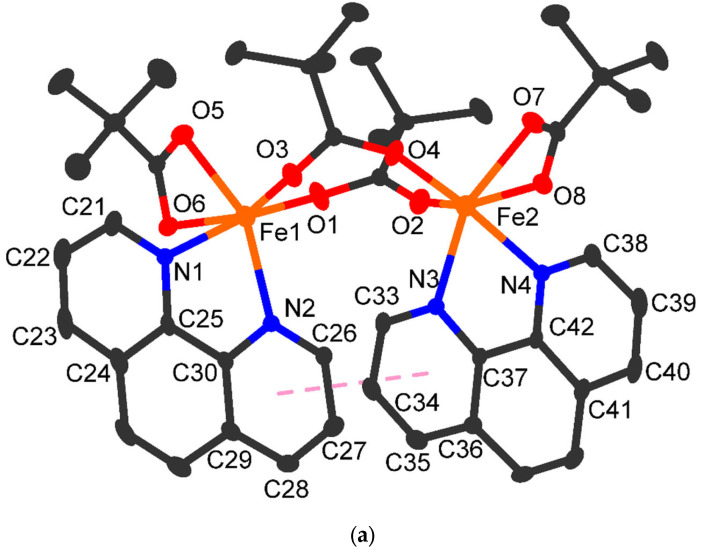

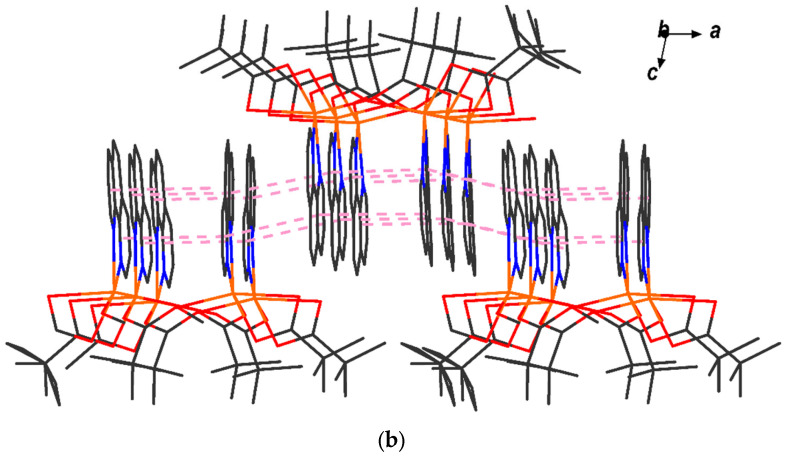
Molecular structure (**a**) and crystal packing (**b**) of **1** ((**a**)—thermal ellipsoids with a probability of 30%; (**a**,**b**)—hydrogen atoms are not shown, π–π contacts are indicated by dotted lines between the centroids of the aromatic rings N2C26–C30 and N3C33–C37).

In the binuclear molecule of complex **1**, the metal atoms (4.568 Å) are connected by two bridging carboxylate groups (Figure 1a, Table 1). The coordination environment of each atom corresponds to a distorted FeN_2_O_4_ octahedron, which is completed by the coordination of the chelate carboxylate group and the chelate 1,10-phenanthroline molecule. Coordinated 1,10-phenanthroline molecules participate in the formation of intra- and intermolecular π–π interactions, which leads to the formation of supramolecular layers parallel to the 0*ab* plane (Figure 1b, Table 2), which contact each other due to van der Waals interactions between *tert*-butyl groups on both sides of the layer. Additionally, the supramolecular layer is strengthened by C–H…O contacts (Table 3). We have found that the stacking of 1,10-phenanthroline ligands and their parallel orientation are possible only in anhydrous media, since the coordination of water molecules and hydrogen bonding interactions between the coordinated water molecules makes π–π interactions less possible [60,61].

**Figure 2:**
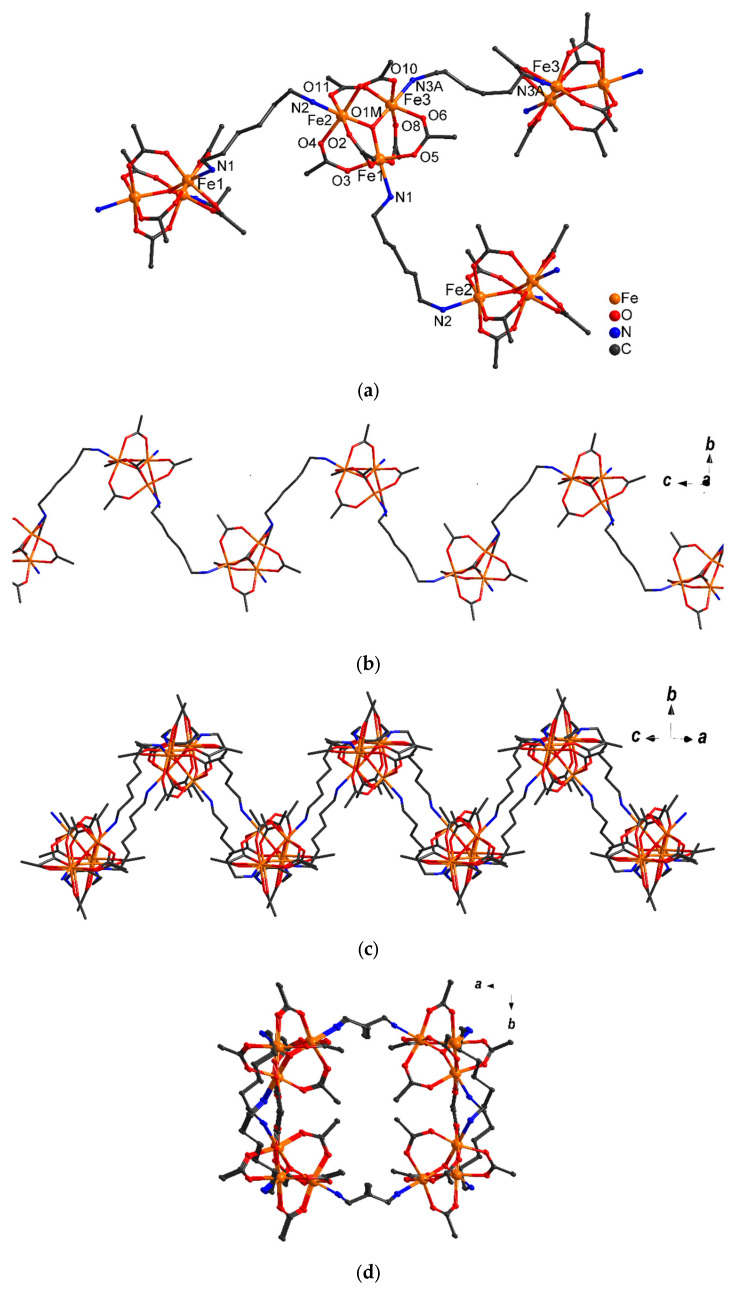
Fragments of 1D polymer structure **2**: (**a**)—binding of triangular fragments {Fe_3_O(piv)_6_} by μ-bridging molecules of 1,6-diaminohexane, (**b**)—fragment of a chain formed by binding {Fe_3_O(piv)_6_} through Fe1 and Fe2 atoms along axes 0*c*, (**c**)—fragment of corrugated tape, (**d**)—projection of the tape packing on the 0*ab* plane (hydrogen atoms and CH_3_ groups are not shown).

Compound **2** is a one-dimensional coordination polymer consisting of triangular fragments {Fe_3_(μ_3_-O)(μ-piv)_6_} and μ-bridged 1,6-diaminohexane molecules (Figure 2a). The structure of {Fe_3_O(piv)_6_} (Table 1) is similar to the known ones in the molecular and polymer complexes [21,62,63]. The difference in the lengths of the Fe-O(μ_3_-O) bonds (Fe(1/3)–O 1.84 Å and Fe(2)–O 2.01 Å) suggests a difference in the oxidation states of iron ions, Fe^III^_2_Fe^II^, which is consistent with the electrical neutrality of the compound and interatomic distances in known compounds with a metal core {Fe^III^_2_M^II^(μ_3_-O)(μ-O_2_CR)_6_} (M^II^ = Mn, Fe, Co, Ni) [2,3,4,26,27]. Early studies on mixed-valence triangular oxo-carboxylate iron(II/III) complexes showed that the Fe-O(μ_3_-O) bond length obtained from X-ray diffraction data determines the oxidation state of the metal ion in the complex (Fe^III^-O(μ_3_-O) are in the range of 1.8–1.9 Å;, the bond length Fe^II^-O(μ_3_-O) is much larger and is close to 2.0–2.1 Å), which is reasonably confirmed by Mössbauer spectroscopy [27,64,65,66]. The coordination polyhedra of FeNO_5_ iron atoms formed by oxo-, carboxylate, and amino groups correspond to a distorted octahedron. The Fe1 and Fe2 atoms of two fragments {Fe_3_O(piv)_6_} (Fe…Fe 9.954 Å) are connected by a bridging diamine molecule (N1-C6-N2), which corresponds to the growth of the chain in the form of a zigzag along the 0*c* axis (Figure 2b). Pairs of Fe3 atoms of two adjacent vertices of a zigzag as part of a pair of chains (Fe…Fe 11.013 Å) are connected by diamine molecules; this leads to the formation of a “corrugated tape (or ladder)” (Figure 2b,c). The nitrogen atoms of the amino groups participate in the formation of H-bonds with the O atoms of the bridging carboxylate groups (Table 3). In the crystal, the chains are laid parallel to each other.

**Figure 3:**
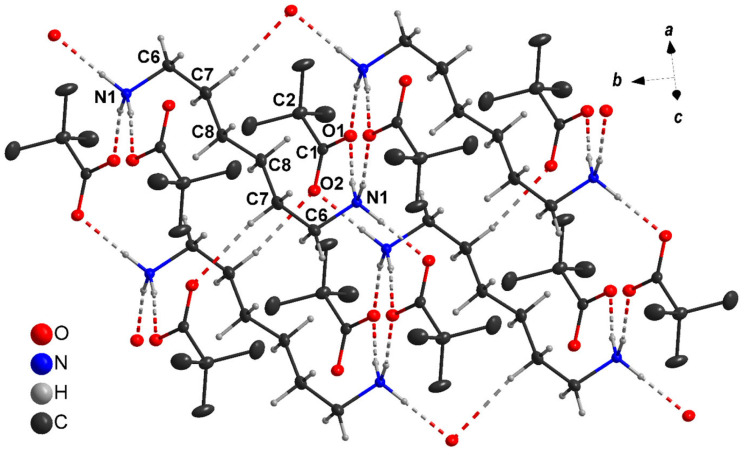
Fragment of the crystal packing of compound **3** (thermal ellipsoids with a probability of 30%, hydrogen atoms at CH_3_ groups are not shown, N–H…O and C–H…O interactions are indicated by the dotted line).

Organic salt **3** consists of pivalic acid anions and 1,6-diaminohexane dications in a ratio of 2:1 (Figure 3). In the crystal, each NH_3_ group participates in the formation of three H-bonds with O atoms of three carboxylate groups (Table 3).

It was previously shown that using the interaction of aliphatic diamines of different lengthsNH_2_(CH_2_)_n_NH_2_ with the same carboxylate anion of triphenylacetic acid leads to the formation of molecular (n = 6) as well as one-dimensional (n = 7–9) and two-dimensional (n = 10–11) coordination polymers [67]. In our case, despite the sterically hindered trimethyl acetate group, the organic salt forms chains due to the branched hydrogen bonds.

Based on the analysis of single-crystal X-ray diffraction data, we can draw a valuable conclusion: we succeeded at obtaining a coordination polymer of iron(II) based on the aliphatic diamine bridging ligand 1,6-diaminohexane for the first time. To the best of our knowledge, prior to this work, such compounds with unambiguously determined molecular and crystal structures were not described.

### 2.3. Powder X-ray Diffraction Analysis

When working with an isolated substance, it is very important to confirm that the entire sample phase corresponds to the data obtained by means of single-crystal X-ray diffraction analysis.

**Figure 4:**
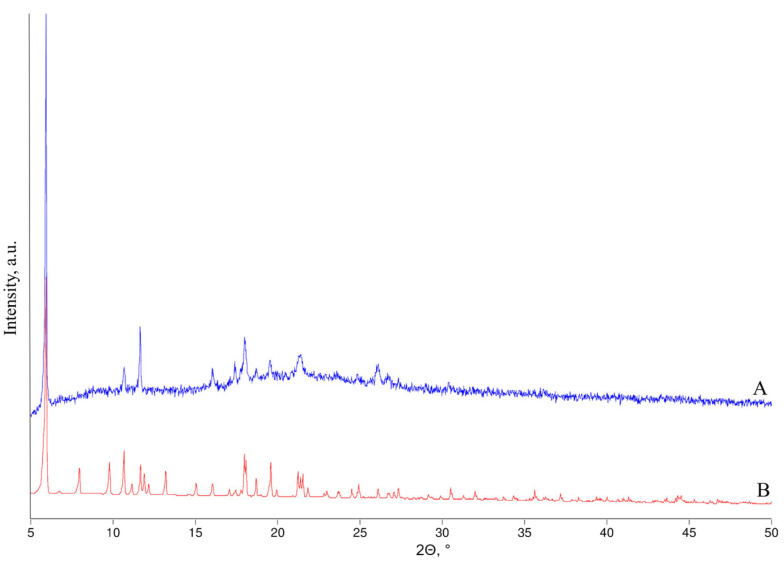
Comparison of experimental (**A**, **blue line**) and theoretical (**B**, **red line**) diffraction patterns for sample **1**.

**Figure 5:**
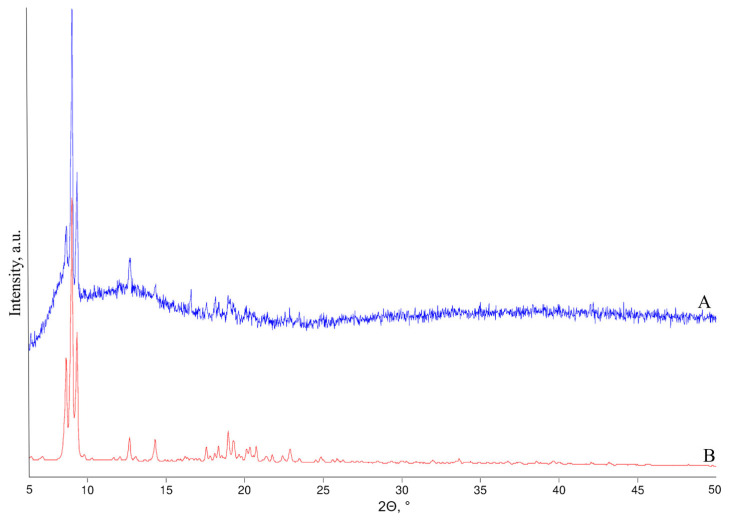
Comparison of experimental (**A**, **blue line**) and theoretical (**B**, **red line**) diffraction patterns for sample **2**.

Powder X-ray diffraction data calculated by the Rietveld method proves the correspondence of the polycrystalline phase to the structure determined by means of single-crystal X-ray diffraction for compounds **1** and **2** (Figure 4 and Figure 5). According to experimental data, complex **1** retains its crystal structure for more than 2 months (Figure S6). After keeping sample **2** in air for 5 h and performing the X-ray diffraction experiment for 8 h (Figure S7), the amorphous component in the diffraction pattern increases. After storing the sample **2** for 2 months, an additional phase related to structure **3** is detected in the sample of complex **2** (Figure S7). Thus, from the experimental diffraction patterns, it follows that the iron(II) complex **1** we obtained in solid form is resistant to moisture and atmospheric oxygen, while complex **2** is exposed to air with the destruction of crystallinity and the formation of organic salt **3**.

### 2.4. Thermal Behavior

**Figure 6:**
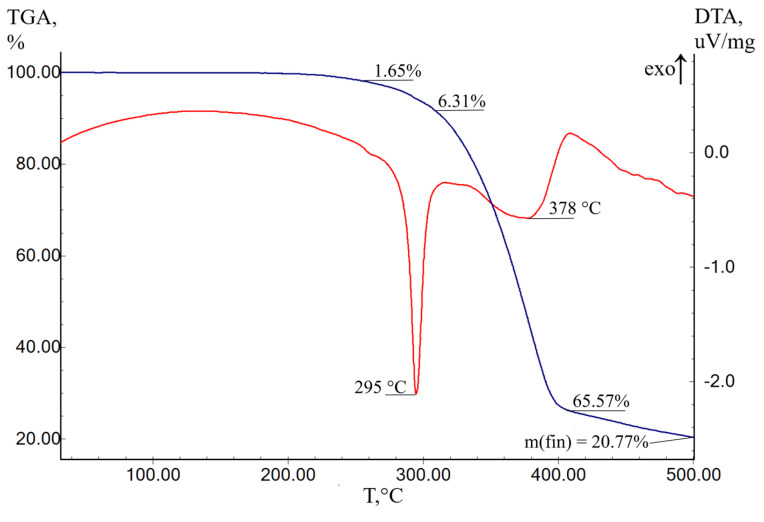
TGA (blue) and DTA (red) curves for complex **1**.

**Figure 7:**
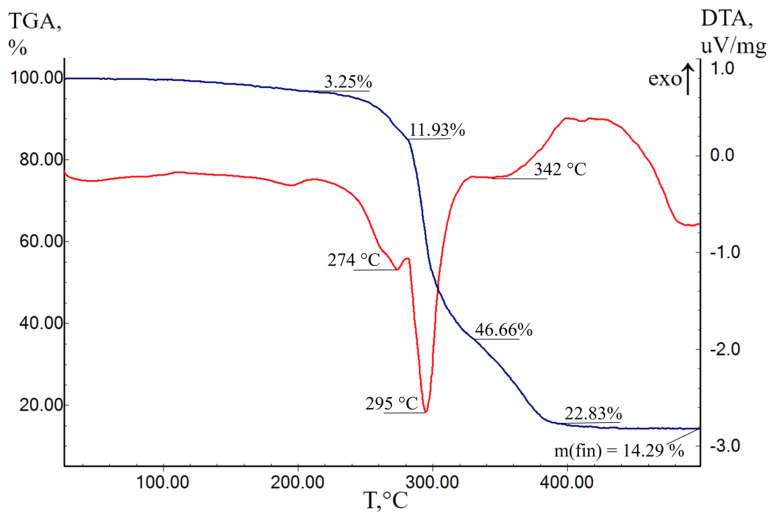
TGA (blue) and DTA (red) curves for complex **2**.

The thermal behavior of complexes **1** and **2** was investigated by simultaneous thermal analysis (STA), a combination of thermogravimetric analysis (TGA) and differential thermal analysis (DTA), in an argon flow (Figure 6 and Figure 7). Complex **1** is stable up to 255 °C (Δ*m* = 1.65%). In the range of 255–308 °C, there is a smooth mass loss (Δ*m* = 6.31%) accompanied by the narrow strong endothermic effect with a peak at 295 °C in the DTA curve, which corresponds to the start of the 1,10-phenanthroline fragment decomposition (*t*_bp_ = 300 °C, Δ*m*_theor_ = 41.07%) and the melting of the thermolysis product (*t*_onset_ = 290 °C). Decomposition of pivalate groups [68] and further destruction and elimination of phen takes place in the range of 308–406 °C (Δ*m* = 65.57%), accompanied by the complex-shaped slight endothermic effect with a peak at 378 °C in the DTA curve. It is worth noting that in the gas-phase mass spectra of trimethylacetate complexes of d-metals at 305–470 °C, the destruction products of trimethyl acetate ions, [C_4_H_9_]^+^, [C_3_H_5_]^+^, [C_2_H_5_]^+^, CH_2_O^+^, and others, are recorded [69]. According to DTG and DTA data, it is not possible to separate the individual stages of decomposition, which overlap each other. In the range of 406–500 °C, further decomposition of the thermolysis products occurs with a weight loss of 6.03%. The residual mass of the sample is higher than theoretically expected for Fe_2_O_3_ and amounts to *m*_exp_ = 20.77%/*m*_theor_ = 18.25% due to the incomplete process of thermal decomposition in an inert atmosphere.

In the case of compound **2,** the initial mass loss **of** 3.25% in the temperature range of 94–212 °C is probably associated with the presence of solvent molecules occluded by the polycrystalline sample. The thermal degradation of organic fragments of complex **2** begins at 212 °C, which is consistent with data known from the previously published papers for complexes with 1,6-diaminohexane [70,71]. In the range of 212–282 °C, there is a smooth weight loss (Δ*m* = 11.93%), accompanied by a weak endothermic effect of the complex form on the DTA curve with a peak at 274 °C, associated with the decomposition and elimination of one 1,6-diaminohexane molecule (*t*_bp_ = 205 °C; Δ*m*_theor_ = 12.03%). The process overlaps with the destruction and elimination of the remaining 0.5 molecule of NH_2_(CH_2_)_n_NH_2_ (Δ*m*_theor_ = 6.02%) and coordinated pivalate anions in the range of 282–332 °C (Δ*m* = 46.66%). Melting of the intermediate thermolysis product [68] appeared on the DTA curve as a strong endothermic effect (*t*_onset_ = 276 °C) with a peak at 295 °C. The decomposition of the remaining fragments of trimethylacetate anions takes place in the range of 332–392 °C, accompanied by a mass loss of 22.83% and a weak endothermic effect on the DTA curve (peak at 342 °C). Above 392 °C, residual weight loss occurs (Δ*m* = 1.18%). The residual mass of the sample is lower than the theoretical one for Fe_2_O_3_ and amounts to *m*_exp_ = 14.29%/*m*_theor_ = 24.9%. The ability of intermediate thermolysis products of trimethyl acetate iron(II,III) complexes to be volatile is consistent with that known from the literature [72] and is confirmed by the presence of a red–brown sediment on the outer surface of the crucible after heating in the case of complexes **1** and **2**.

Thus, thermal studies of new iron complexes showed that molecular complex **1** is stable up to 255 °C, while coordination polymer **2** begins to decompose at 212 °C. Both complexes **1** and **2** demonstrate volatility and melting of the intermediate thermolysis products with a peak on the DTA curve at 295 °C.

## 3. Materials and Methods

### 3.1. General Remarks

The synthesis of the compounds **1**–**3** was carried out using a modified Schlenk technique. Photographs of the main stages of synthesis are available in the Supplementary Materials (Figures S4 and S5). Acetonitrile CH_3_CN (Khimmed, reagent-grade, Russia) was dried over phosphorus(V) oxide, distilled, and stored in an evacuated glass flask with activated molecular sieves (3 Å), from where it was taken by condensation into a chemical reactor (glass ampule with a Teflon stopcock) immediately before synthesis; ethanol EtOH (95%, Ferein, Russia) was degassed and taken by condensation into the chemical reactor. Iron sulfate heptahydrate FeSO_4_∙7H_2_O (Khimmed, reagent grade, Russia), 1,10-phenanthroline monohydrate (Sigma-Aldrich, pure grade, Steinheim, Germany), and 1,6-diaminohexane (Merck, analytical grade, Germany) were used without additional purification. Potassium pivalate (Kpiv) was obtained according to a known synthetic procedure [73]. Before reacting with iron(II) sulfate, potassium pivalate was heated at 140 °C in an oil bath for 24 h in a dynamic vacuum. Iron(II) pivalate [Fe(piv)_2_]_n_ was obtained by an exchange reaction from FeSO_4_∙7H_2_O and Kpiv [5]. Iron pivalate is poorly soluble in ethanol and acetonitrile; it forms a gel-like mass. Adding excess pyridine or an equimolar amount of 1,10-phenanthroline converts iron(II) pivalate into soluble complexes of the composition [Fe_2_(piv)_4_(py)_2_] [74] or [Fe_2_(piv)_4_(phen)_2_] (current work), which allows filtration from insoluble potassium sulfate.

Simultaneous thermal analysis (STA), a combination of thermogravimetric analysis (TGA) and differential thermal analysis (DTA), was performed for **1** and **2** on a synchronous thermal analyzer DTG–60 (Shimadzu, Kyoto, Japan) in an argon flow (100 mL/min) at a speed of 10 °C/min in the range of 23–500 °C. The measurements were carried out in Al crucibles with a hole. The mass of the samples was 4.18–4.60 mg.

IR spectra of the compounds were recorded in the range of 400–4000 cm^–1^ on a Perkin Elmer Spectrum 65 spectrophotometer (Waltham, MA, USA) equipped with a Quest ATR Accessory (Specac, Orpington, UK) using the attenuated total internal reflection (ATR) method. Elemental analysis was performed on an automatic CHNS analyzer “EuroEA–3000” (EuroVektor, Pavia, Italy).

X-ray powder diffraction analysis of samples **1** and **2** was carried out on a Bruker D8 Advance diffractometer (Bruker AXS, Madison, WI, USA) (step: 0.02° 2θ; interval: 5–50° 2θ) (CuKα, λ = 1.54060 Å, Ni filter, LYNXEYE detector, reflection geometry) at 20 °C. Comparisons of experimental and theoretical diffraction patterns, as well as modeling of the calculated curve, were carried out in the TOPAS 4 software program package using structures of the compounds, obtained from low-temperature single-crystal diffraction data.

X-ray structural analysis of single crystals of the studied compounds was carried out on a Bruker D8 Venture automatic diffractometer (CCD detector, Mo*K*_α_/Cu*K*_α_, graphite monochromator) (Bruker AXS, Madison, WI, USA). A semi-empirical correction for absorption was used [75]. The structure was solved by the direct and Fourier methods and refined in the full-matrix anisotropic approximation for all non-hydrogen atoms. Hydrogen atoms at carbon atoms of organic ligands are generated geometrically and refined in the “rider” model. Calculations were carried out using the SHELXL 2018/3 software package [76] using the OLEX2 software package [77]. The structure of **1** was refined, taking into account the disorder of the *tert*-butyl group. The structure of **2** was refined, taking into account the disorder of *tert*-butyl groups and 1,6-diaminohexane using standard constraints (ISOR, DFIX, RIGU, EADP). The structure of **3** was solved, taking into account twinning (twin -1 0 0 0 -1 0 0 0 -1, Flack parameter is 0.113(18)). Crystallographic parameters and structure refinement details are given in Table 4. Atomic coordinates, thermal parameter values, and a list of all reflections are deposited in the Cambridge Structural Data Bank (CCDC 2336889–2336891 for **1**–**3,** respectively) and can be obtained upon request to deposit@ccdc.cam.ac.uk or http://www.ccdc.cam.ac.uk/structures (accessed on 4 March 2024).

### 3.2. Synthesis of [Fe_2_(piv)_4_(Phen)_2_] (***1***)

To the iron(II) pivalate prepared in situ from FeSO_4_∙7H_2_O (0.278 g, 1.0 mmol) and Kpiv (0.280 g, 2.0 mmol), 10 mL of acetonitrile was condensed and added 1,10–phenanthroline monohydrate (0.198 g, 1 mmol). The red–colored reaction mixture was filtered to remove potassium sulfate. The filtrate was sealed in a glass ampoule, heated in an oil bath at a temperature of 120 °C for 10 h (caution! high pressure), further cooling to room temperature led to the formation of blue–green crystals. The yield of **1** is 0.341 g (78%). The structure of the complex was determined with single crystal X-ray diffraction analysis. The phase purity of sample **1** was determined by X-ray powder diffraction analysis. Calc. for C_44_H_52_Fe_2_N_4_O_8_ (%): C, 60.29; H, 5.98; N, 6.39. Found (%): C, 60.25; H, 5.91; N, 6.24. IR (ATR, ν, cm^–1^): 3060 w, 3017 w, 2951 m, 2901 w, 2863 w, 1579 m, 1536 w, 1478 m, 1413 vs, 1356 m, 1222 s, 1144 w, 1102 w, 1032 w, 985 w, 940 w, 890 m, 848 m, 790 m, 722 m, 635 w, 601 m, 556 w, 535 w, 504 w, 477 w.

### 3.3. Synthesis of [Fe_3_O(piv)_6_(dahx)_1.5_]_n_ (***2***)

A solution of 1,6–diaminohexane (0.130 g, 1.12 mmol) in 2 mL of anhydrous MeCN was added to 1 mmol of complex **1** prepared in situ in 10 mL of acetonitrile. The reaction mixture was sealed in a glass ampoule and heated in an oil bath at 100 °C for 5 h until a solution was formed (*caution! high pressure*). Cooling to room temperature (24 °C) led to the formation of red crystals of **2** (0.122 g, the yield is 38% per 1 mmol of **1**) and white crystals of (H_2_dahx)(piv)_2_ (**3**). Separation of **2** and **3** was carried out by multiple decantation. The structure of the compounds was determined by single-crystal X-ray diffraction analysis. The phase purity of sample **2** was determined by X-ray powder diffraction analysis. Calc. for C_39_H_78_Fe_3_N_3_O_13_ (%): C, 48.56; H, 8.15; N, 4.36. Found (%): C, 48.03; H, 8.24; N, 4.21.

IR of **2** (ATR, ν, cm^–1^): 3370 w, 3263 w, 3134 w, 2958 m, 2924 w, 2861 w, 1690 w, 1596 m, 1550 w, 1480 m, 1413 s, 1357 m, 1221 m, 1002 m, 890 m, 788 w, 709 w, 661 w, 597 s, 524 w, 417 vs.

IR of **3** (ATR, ν, cm^–1^): 2948 m, 2861 w, 2656 w, 2574 w, 2173 w, 1630 m, 1525 vs, 1482 s, 1402 s, 1357 s, 1325 w, 1219 s, 1154 w, 1089 w, 1001 w, 882 m, 838 w, 792 m, 728 w, 590 m, 532 s, 433 w.

## 4. Conclusions

Thus, a new 1D polymer **2** based on the triangular oxo-carboxylate moiety of {Fe_3_O(O_2_CR)_6_} and aliphatic 1,6-diaminohexane was prepared. The resulting 1D polymer **2** has a corrugated tape topology in which two polymer chains {Fe_3_(μ_3_-O)(piv)_6_(dahx)}_n_ are linked by bridging diamine molecules. In fact, obtaining this complex is determined by chance. Currently, our attempts to isolate the reaction product of iron(II) pivalate and 1,6-diaminohexane have been confronted with difficulties to isolate the product from a mixture of different polycrystals. The use of the new complex **1** as a starting reagent that is soluble in acetonitrile did not lead to the isolation of a polymer product, which contains carboxylate anions, 1,10-phenatroline and 1,6-diaminohexane, but, as we assume, traces of atmospheric oxygen led to the rearrangement of the dimer iron(II) into a oxo-carboxylate fragment {Fe_3_(μ_3_-O)(piv)_6_} and iron complex(es) with 1,10-phenanthroline. Note that we were previously unable to isolate such a complex from iron(III) oxo-carboxylates due to the presence of oxygen-containing ligands in the coordination environment of oxophilic metal atoms, but this became possible against the background of their deficiency in the reaction mixture. This unpredictable result showed that the production of iron complexes based on oxo-carboxylate fragments and polyamines is possible through the synthesis of ferrous iron complexes and the generation of {Fe_3_O(O_2_CR)_6_} under conditions of deficiency of O-containing ligands.

## Figures and Tables

**Table 1:** Main bond lengths (Å) and angles (degrees) in the coordination polyhedra of compounds **1** and **2**.

1					
Fe1-O1	2.037 (3)	Fe2-O2	2.059 (3)	O-Fe1-O	59.30 (11), 88.69 (12)–99.42 (13)
Fe1-O3	2.064 (3)	Fe2-O4	2.050 (3)	O-Fe1-N	85.86 (11)–101.62 (13)
Fe1-O5	2.100 (3)	Fe2-O7	2.119 (3)	N-Fe1-N	76.26 (12)
Fe1-O6	2.321 (3)	Fe2-O8	2.310 (3)	O-Fe2-O	59.52 (11), 94.44 (15)–100.51 (13)
Fe1-N1	2.175 (3)	Fe2-N3	2.179 (3)	O-Fe2-N	87.17 (12)–102.18 (13)
Fe1-N2	2.173 (3)	Fe2-N4	2.175 (3)	N-Fe2-N	76.15 (12)
**2**					
Fe-O(μ_3_-O)	1.831 (7), 1.840 (6), 2.009 (7)	Fe-N	2.177 (10), 2.180 (9), 2.068 (14)	O-Fe-O	85.1 (3)–98.0 (3)
Fe-O(O_2_CR)	1.994 (8)–2.133 (8)	Fe-O(μ_3_-O)-Fe	118.2 (3)–123.2 (4)	O-Fe-N	80.8 (3)–83.1 (3)

**Table 2:** Parameters of π⋅⋅⋅π interactions in crystal packing of 1 (Cg is the centroid of the phenyl ring; Cg-Perp is the shortest distance from Cg to the plane of the neighboring cycle; α is the angle between the planes of Cg).

Interaction	Cg⋅⋅⋅Cg, Å	Cg⋅⋅⋅Perp, Å	α, deg
Cg_1_(N2C26-C30)…Cg_3_(N3C33-C37)	3.673 (3)	3.4 (2)	3.4597 (18)
Cg_1_…Cg_2_(N1C21–C27) [1 − *x*, 1/2 + *y*, 1 − *z*]	3.655 (3)	1.4 (2)	3.4027 (18)
Cg_2_…Cg_4_(N4C38–C42) [*x*, 1 + *y*, *z*]	3.562 (3)	4.2 (2)	3.2932 (18)

**Table 3:** Parameters of hydrogen bonds in crystals **1**–**3**.

Hydrogen Bond	D–H, Å	H⋅⋅⋅A, Å	D⋅⋅⋅A, Å	D–H⋅⋅⋅A, deg
**1**				
C19-H19C…O7	0.98	2.46	2.850 (11)	103
C31-H31…O6 [1 − *x*, 1/2 + *y*, 1 − *z*]	0.95	2.34	3.224 (6)	154
C44-H44…O8 [2 − *x*, −1/2 + *y*, 1 − *z*]	0.95	2.40	3.284 (6)	156
**2**				
N1-H1B…O7	0.91	2.41	2.732 (13)	100
N2-H2AB…O9 [*x*, 1 − *y*, −1/2 + *z*]	0.91	2.42	2.798 (13)	103
C8-H8A…O4	0.98	2.59	2.922 (14)	100
C10-H10C…O4	0.98	2.42	2.797 (16)	103
C15-H15C…O6	0.98	2.49	2.870 (17)	103
C28-H28A…O12		2.56	2.898 (14)	100
**3**				
N1-H1A…O1 [2 − *x*, 1 − *y*, 1 − *z*]	0.91	1.90	2.7762 (15)	162
N1-H1B…O1	0.91	1.90	2.7954 (14)	170
N1-H1C…O2 [1 − *x*, 1 − *y*, 1 − *z*]	0.91	1.81	2.7134 (14)	172
C7-H7A…O2 [1 − *x*, 2 − *y*, 1 – *z*]	0.99	2.50	3.4580 (17)	163

**Table 4:** Crystallographic data, details of the data collection, and characteristics of the data refinement for **1**–**3**.

Parameter	Value		
	1	2	3
Molecular formula	C_44_H_52_Fe_2_N_4_O_8_	C_39_H_78_Fe_3_N_3_O_13_	C_16_H_36_N_2_O_4_
*M*_r_	876.59	964.59	320.47
*T* (K)	150	100	150
λ (Å)	0.71073	1.54178	0.71073
Space group	*P*2_1_	*Pbcn*	*P*–1
*a* (Å)	13.573 (3)	19.0022 (5)	6.2580 (16)
*b* (Å)	10.069 (4)	27.4782 (7)	7.0260 (19)
*c* (Å)	15.668 (4)	19.9912 (5)	11.487 (2)
α (°)	90	90	82.544 (7)
β (°)	101.762 (8)	90	89.035 (9)
γ (°)	90	90	83.774 (9)
*V* (Å^3^)	2096.3 (11)	10438.3 (5)	497.9 (2)
*Z*	2	8	1
*ρ*_calc_ (g·cm^−3^)	1.228	1.389	1.069
µ (mm^−1^)	0.750	7.053	0.075
Crystal size (mm)	0.25 × 0.25 × 0.04	0.12 × 0.10 × 0.08	0.25 × 0.20 × 0.10
*T*_min_, *T*_max_	0.6355, 0.7465	0.4814, 0.7489	0.6538, 0.7460
*θ* (^o^)	2.22–33.15	2.83–45.14	3.28–29.97
Number of reflections collected	24,319	29,639	3576
Number of unique reflections	13,961	4226	2313
Number of reflections with *I* > 2σ(*I*)	12,059	2891	1907
*R*_int_	0.0298	0.0399	0.0229
*R*_1_/*wR*(*F*^2^), [*I* > 2*σ*]	0.0602/0.1435	0.0715/0.1924	0.0430/0.1092
*R*_1_/*wR*(*F*^2^), (for all reflections)	0.0717/0.1516	0.1053/0.2170	0.0522/0.1154
*S*	1.095	1.061	1.026
Parameters	567	671	104
Δρ_max_/Δρ_min_ (e Å^−3^)	2.858/−0.485	0.442/−0.309	0.287/−0.151
X-ray source	MoKα, 0.71073 Å	CuKα, 1.54178 Å	MoKα, 0.71073 Å

## Data Availability

The structure parameters of the obtained compound were deposited into the Cambridge Structural Database (CCDC Nos. 2336889 (**1**), 2336890 (**2**), 2336891 (**3**)) deposit@ccdc.cam.ac.uk or http://www.ccdc.cam.ac.uk/data_request/cif (accessed on 4 March 2024).

## Supplementary Materials

The following supporting information can be downloaded at https://www.mdpi.com/article/10.3390/molecules29092125/s1: Figures S1–S3: IR spectra of compounds **1**–**3;** Figure S4: Graphical information of the synthetic procedure to obtain compound [Fe_2_(piv)_4_(phen)_2_] **1**; Figure S5: Graphical information of the synthetic procedure to obtain compound [Fe_3_O(piv)_6_(dahx)_1.5_]*_n_* (**2**) and **3**; Figure S6: Comparison of experimental (A, black line, 26 March 2024; B, blue line, 12 January 2024) and theoretical (C, red line) diffraction patterns for sample **1**; Figure S7: Comparison of experiments for sample **2** over time in air atmosphere (blue line (B)—after 40 min in air; black line (A)—after 13 h in air) and theoretical diffraction patterns for **2** (red line (C)) and 3 (pink line (D)); Figure S8: Comparison of experimental for sample **2** over time in air atmosphere (blue line (B)—after 40 min in air; black line (A)—after 2 months in air) and theoretical diffraction patterns for **2** (red line (C)) and **3** (pink line (D)).
