# Supplementary material for: Coordination Polymer Based on a Triangular Carboxylate Core {Fe(μ3-O)(μ-O2CR)6} and an Aliphatic Diamine
Source: Molecules. 2024 May 3;29(9):2125. doi: 10.3390/molecules29092125 (PMC11085704; doi:10.3390/molecules29092125)
Supplement: Supplementary file 1 [file molecules-29-02125-s001.zip › molecules-2986555-supplementary.pdf]

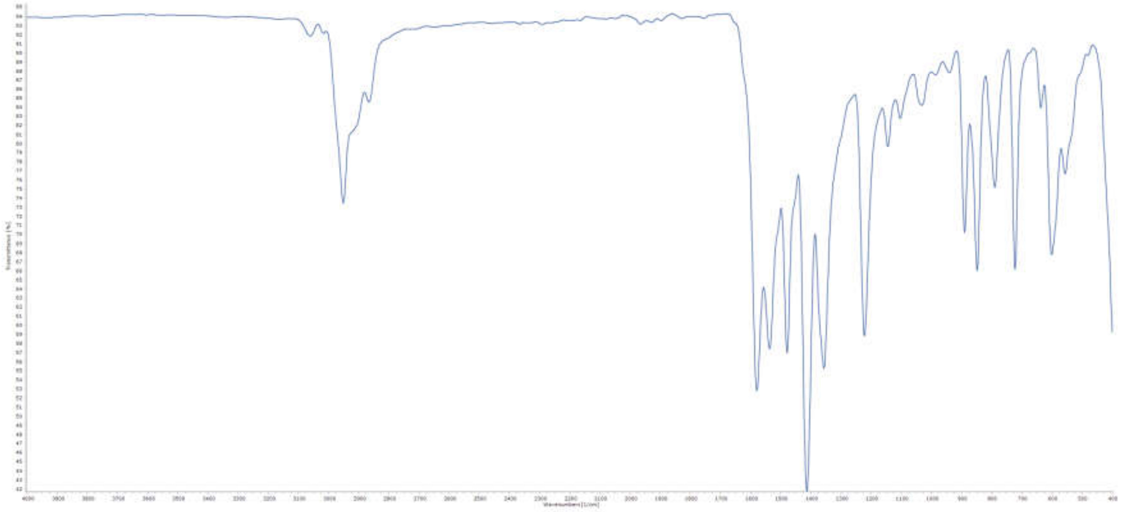

Figure S1. IR-Spectra of **1**

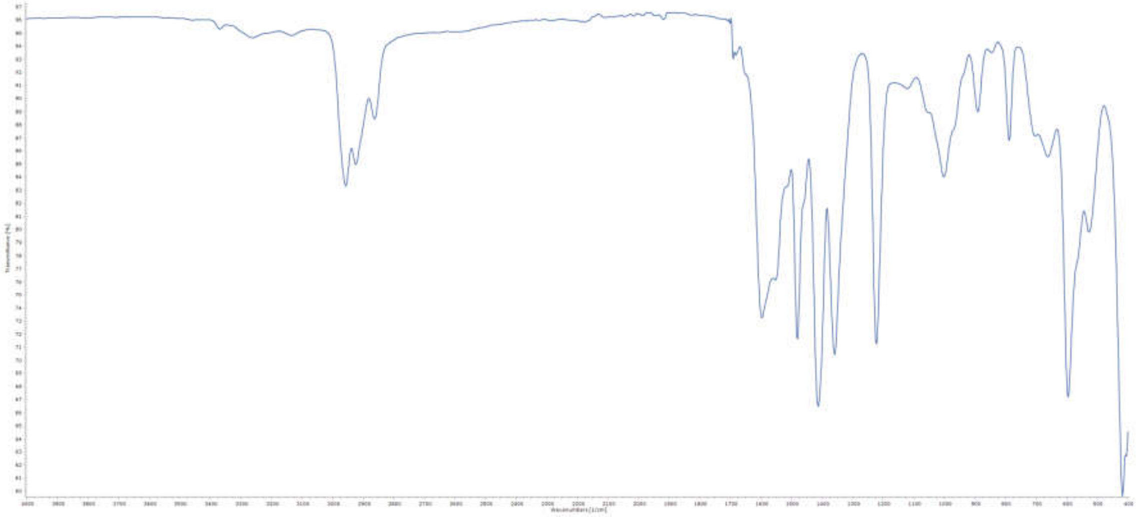

Figure S2. IR-Spectra of **2**

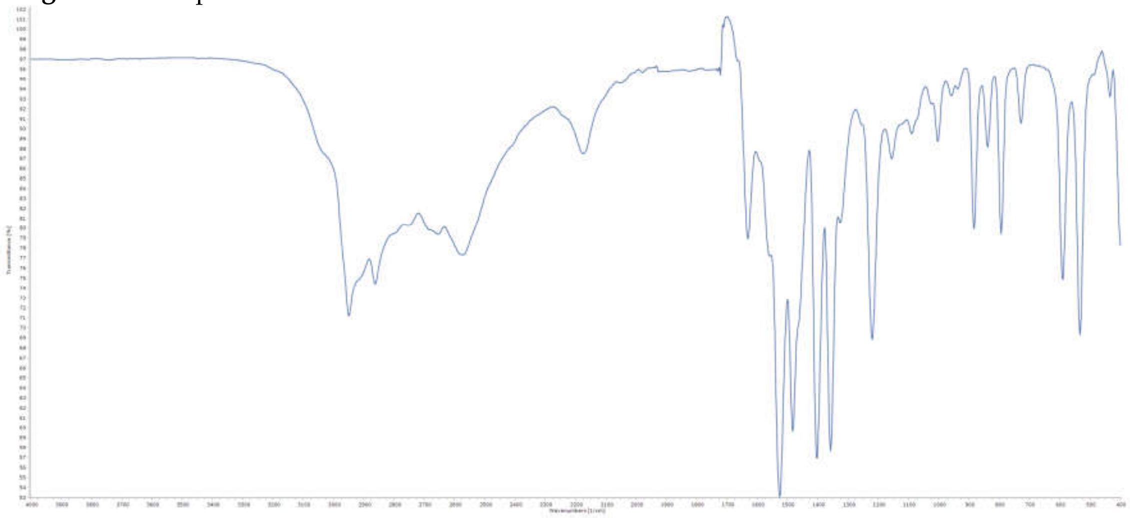

Figure S3 IR-Spectra of **3**

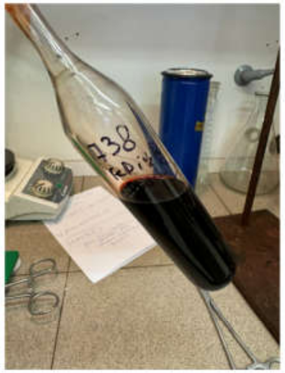

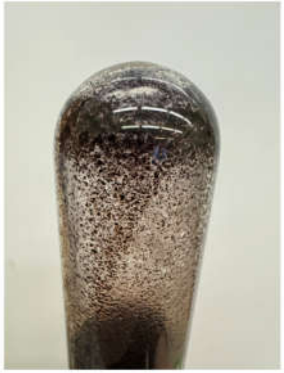

To the iron(II) pivalate prepared in situ from $FeSO_4{\cdot}7H_2O$ (0.278 g, 1.0 mmol) and Kpiv (0.280 g, 2.0 mmol), 10 ml of acetonitrile was condensed and added 1,10-phenanthroline monohydrate (0.198 g, 1 mmol)

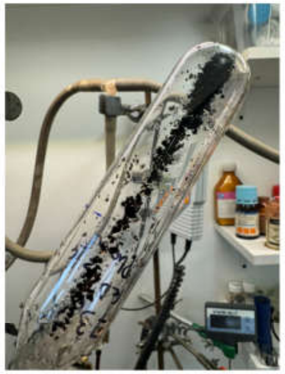

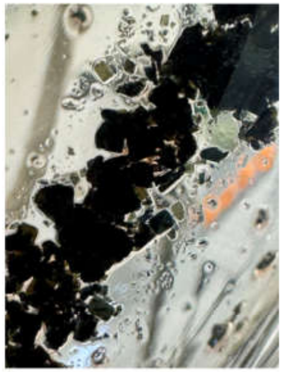

The filtrate was sealed in a glass ampoule, heated in an oil bath at a temperature of 120 ° C for 10 hours, further cooling to room temperature led to the formation of blue-green crystals

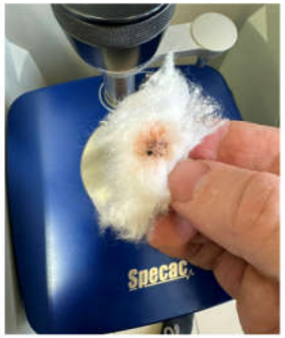

In air, blue-green crystals turn orange when exposed to acetone. However, powder in air without the action of a solvent is resistant to atmospheric oxygen for more than two months (see Figure S6)

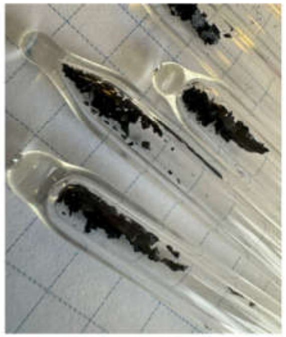

Isolated single crystals in a sealed glass ampoules

Figure S4. Synthesis of **compound [Fe_2(piv)$_4$(phen)$_2$] 1**

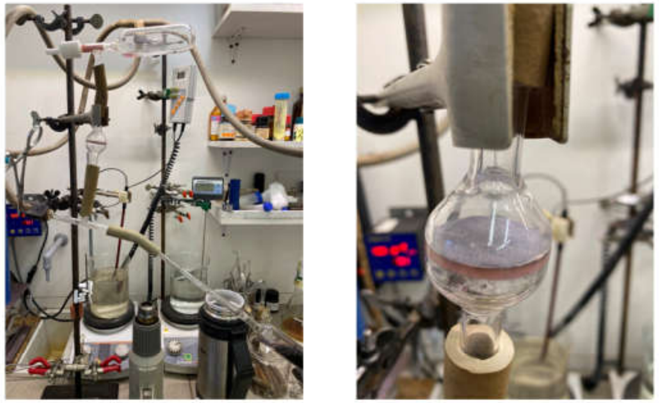

To the iron(II) pivalate prepared in situ from $FeSO_4{\cdot}7H_2O$ (0.278 g, 1.0 mmol) and Kpiv (0.280 g, 2.0 mmol), 10 ml of acetonitrile was condensed and added 1,10-phenanthroline monohydrate (0.198 g, 1 mmol). The red-colored reaction mixture was filtered from potassium sulfate.

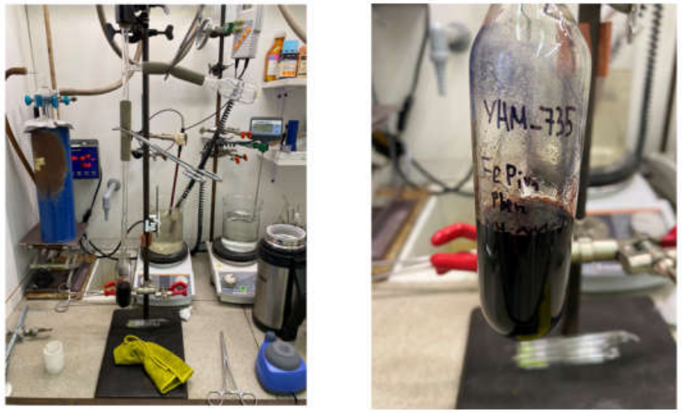

A solution of 1,6-diaminohexane (0.130 g, 1.12 mmol) in 2 ml of anhydrous CH_3CN was added to 1 mmol of complex I prepared *in situ* in 10 ml of acetonitrile. The reaction mixture was sealed in a glass ampoule and heated in an oil bath at 100 ° C for 5 hours until a solution was formed (*caution! high pressure*)

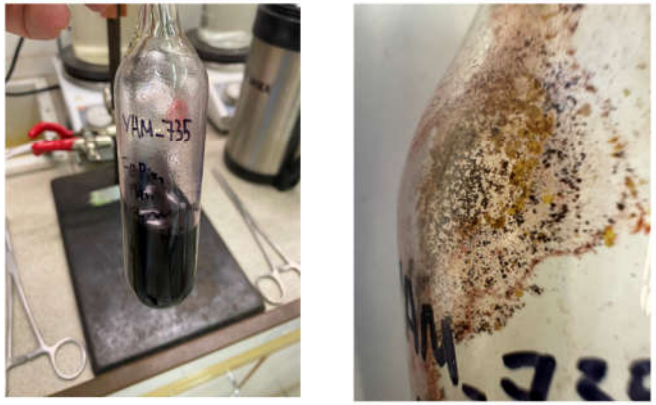

Cooling to room temperature (24 ° C) led to the formation of red (**2**, 0.122 g, 38% per 1 mmol **1**) and white crystals ((piv)2(H_2hmda), **3**).

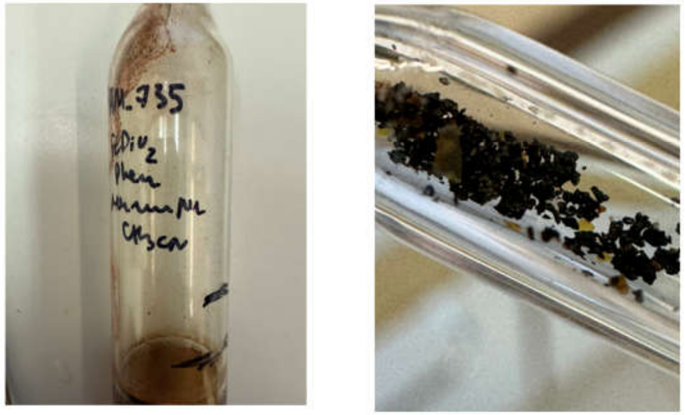

Isolated single crystals in a sealed glass ampoule. Separation of **2** and **3** was carried out by multiple decantation

Figure S5. Synthesis of compound **$[Fe_3O(Piv)_6(NH_2(CH_2)_6NH_2)_{1.5}]_n$ (2) + 3**

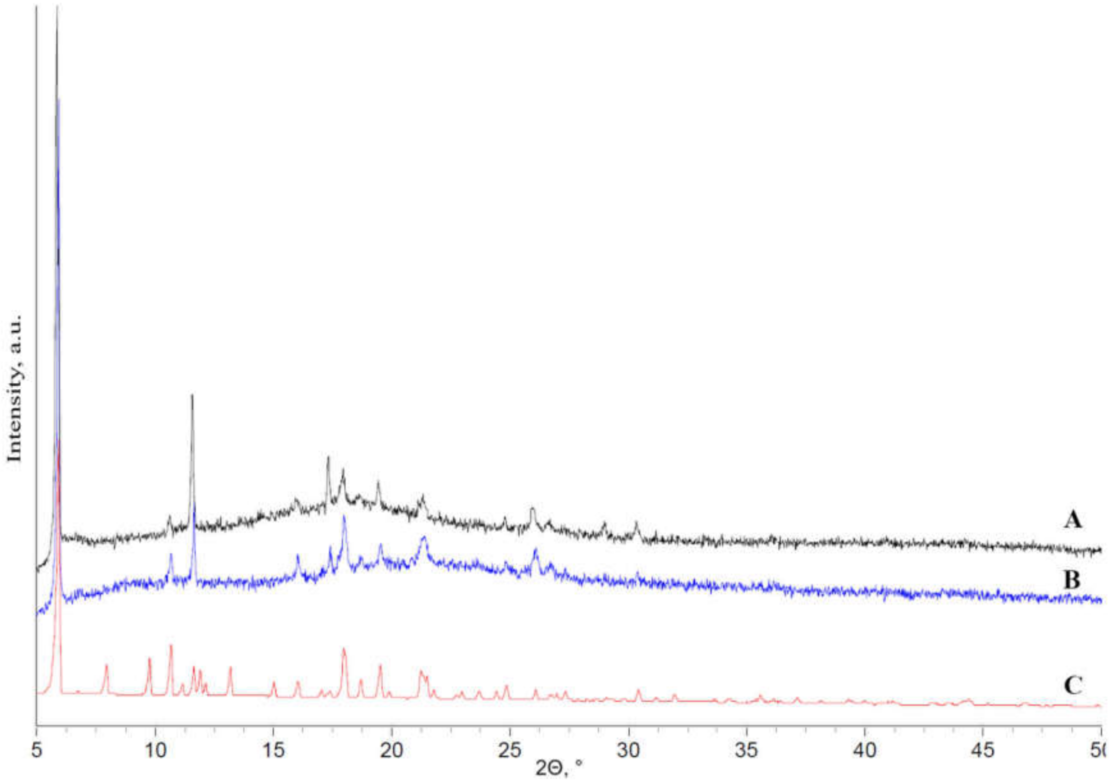

Figure S6. Comparison of experimental (A, black line 26 March 2024; B, blue line, 12 January 2024) and theoretical (C, red line) diffraction patterns for sample **1**

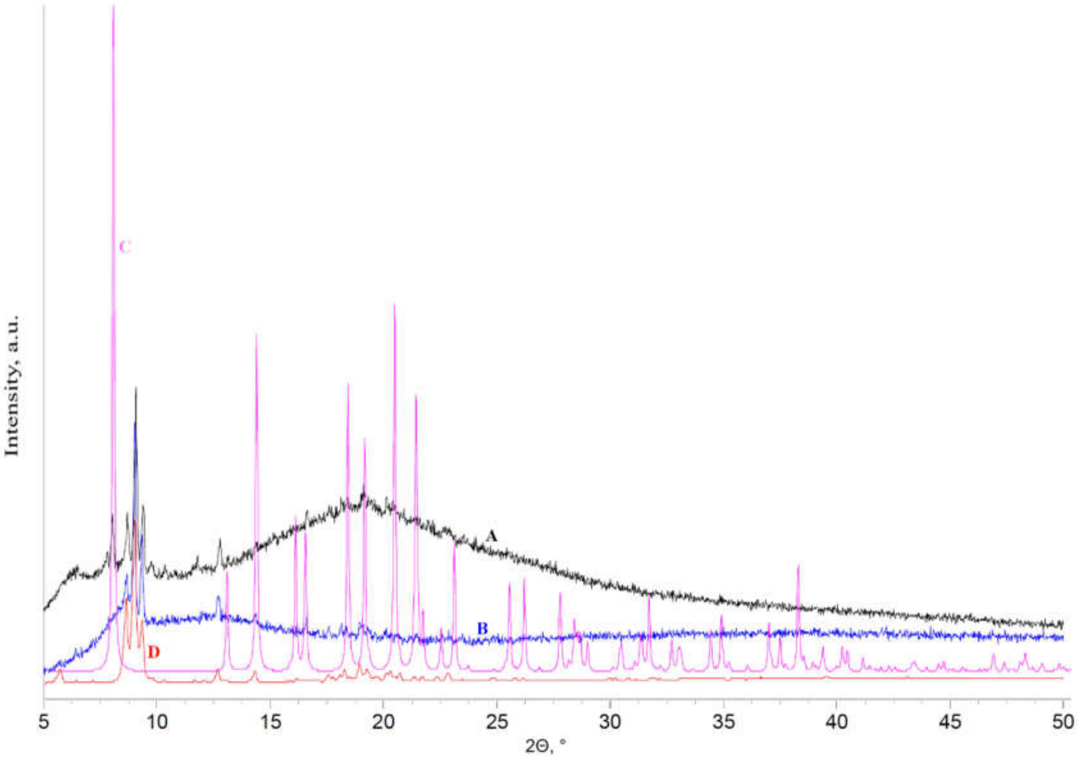

Figure S7. Comparison of experimental for sample **2** in time in air atmosphere (blue line (B) - after 40 min in air; black line (A) - after 13 h in air) and theoretical diffraction patterns for **2** (red line (C)) and 3 (pink line (D)).

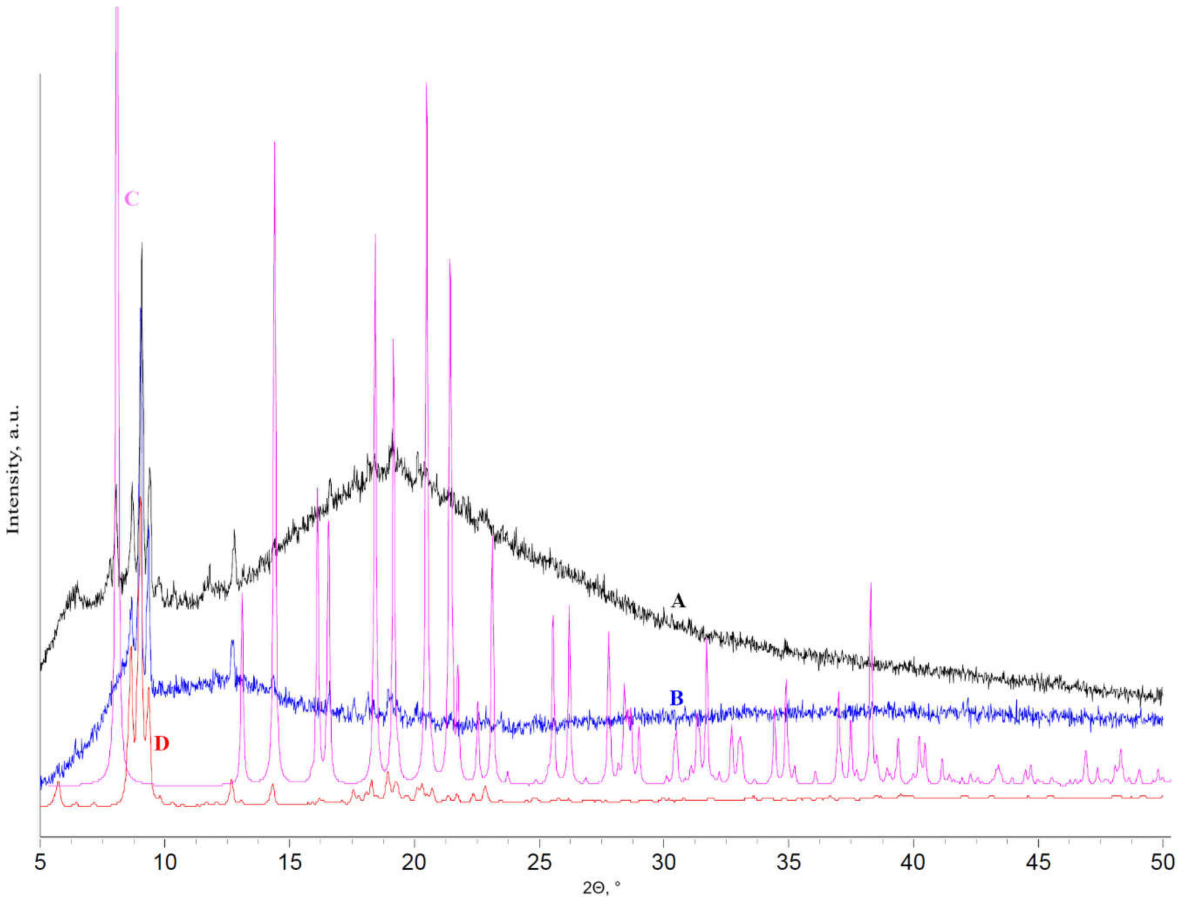

Figure S8. Comparison of experimental for sample **2** in time in air atmosphere (blue line (B) - after 40 min in air; black line (A) - after 2 months in air) and theoretical diffraction patterns for **2** (red line (C)) and **3** (pink line (D)).
